# Neutralization sensitivity of HIV-1 subtype B’ clinical isolates from former plasma donors in China

**DOI:** 10.1186/1743-422X-10-10

**Published:** 2013-01-05

**Authors:** Yabo OuYang, Jianping Sun, Yang Huang, Lu Lu, Weisi Xu, Xintao Hu, Kunxue Hong, Shibo Jiang, Yiming Shao, Liying Ma

**Affiliations:** 1 State Key Laboratory for Infection Disease Prevention and Control, National Center for AIDS/STD Control and Prevention (NCAIDS), Chinese Center for Disease Control and Prevention (China-CDC), 102206, Beijing, China; 2 Key Laboratory of Medical Molecular Virology of Ministries of Education and Health, Shanghai Medical College and Institute of Medical Microbiology, Fudan University, 200032, Shanghai, China; 3 Lindsley F. Kimball Research Institute, New York Blood Center, 10065, New York, NY, USA

**Keywords:** HIV-1 subtype B’, Clinical isolates, Neutralization sensitivity

## Abstract

**Background:**

HIV-1 subtype B’ isolates have been predominantly circulating in China. Their intra- and inter-subtype neutralization sensitivity to autologous and heterologous plasmas has not been well studied.

**Results:**

Twelve HIV-1 B’ clinical isolates obtained from patients were tested for their intra- and inter-subtype neutralization sensitivity to the neutralization antibodies in the plasmas from patients infected by HIV-1 B’ and CRF07_BC subtypes, respectively. We found that the plasmas from the HIV-1 B’-infected patients could potently neutralize heterologous viruses of subtype B’ with mean ID50 titer (1/x) of about 67, but they were not effective in neutralizing autologous viruses of subtype B’ with mean ID50 titer (1/x) of about 8. The plasmas from HIV-1 CRF07_BC-infected patients exhibited weak inter-subtype neutralization activity against subtype B’ viruses with ID50 titer (1/x) is about 22. The neutralization sensitivity of HIV-1 B’ isolates was inversely correlated with the neutralizing activity of plasmas from HIV-1 B’-infected patients (Spearman’s *r* = −0.657, *P* = 0.020), and with the number of potential N-glycosylation site (PNGS) in V1-V5 region (Spearman’s *r* = −0.493, *P* = 0.034), but positively correlated with the viral load (Spearman’s *r* = 0.629, *P* = 0.028). It had no correlation with the length of V1-V5 regions or the CD4+ T cell count. Virus AH259V has low intra-subtype neutralization sensitivity, it can be neutralized by 17b (IC_50_: 10μg/ml) and 447-52D (IC_50_: 1.6μg/ml), and the neutralizing antibodies (nAbs) in plasma AH259P are effective in neutralizing infection by the primary HIV-1 isolates with different subtypes with ID50 titers (1/x) in the range of 32–396.

**Conclusions:**

These findings suggest that the HIV-1 subtype B’ viruses may mutate under the immune pressure, thus becoming resistant to the autologous nAbs, possibly by changing the number of PNGS in the V1-V5 region of the viral gp120. Some of primary HIV-1 isolates are able to induce both intra- and inter-subtype cross-neutralizing antibody responses.

## Background

The HIV-1 epidemic remains unchecked in China. The HIV-1 subtype B’ (Thailand variant of subtype B, also known as Thai B [1,2]), is one of the predominant subtypes circulating in former plasma donors (FPDs) who were infected by HIV-1 in the contaminant plasma, mainly in four provinces (Henan, Anhui, Hubei and Shanxi) of China [3,4]. HIV-1 subtype B’ was divergent and formed a monophyletic group which can be distinguished from the B subtype found in North America, Europe and elsewhere in the world. Some distinct signature mutation sites between B’ and typical B subtype were found around the p17 and V3 regions [1,5,6]. Previously, the HIV-1 CRF07_BC recombinant was mainly circulating among the intravenous drug users (IDUs) in Xinjiang Uyghur Autonomous Region of China and later spread throughout the entire country [3,7]. CRF07_BC is derived from subtype B’ and Indian subtype C lineages [8,9].

Neutralizing antibodies (nAbs) are likely to be a critical component of protective immunity if a human immunodeficiency virus type 1 (HIV-1) vaccine is to be effective [10]. Most individuals developed nAbs to their own virus (autologous neutralization) within a few months of infection [11], whereas plasmas from chronically-infected HIV-1 subjects display various degrees of cross-reactive neutralizing activities [12,13]. The development of cross-reactive nAbs during HIV-1 infection correlates with duration of infection, higher plasma viral RNA load and lower CD4^+^ T cell counts [13-15].

NAbs could not eradicate the invasive virions because of the emergence of viral mutants resistant to autologous nAbs, even while retaining their ability to neutralize heterologous viruses of the same subtype (heterologous neutralization) [16,17]. Some studies revealed the mechanisms that HIV-1 escaped from nAbs through extensions of the variable loops of Env, protection of conserved regions from antibody recognition, extensive glycosylation patterns of Env, steric occlusion and conformational masking of receptor binding sites [10,16,18-20].

Envelope glycoprotein of HIV-1 is the target of nAbs, HIV-1 primary isolates can evolve over time to escape from autologous neutralization by increasing the level and/or changing the pattern of glycosylation on the viral *env*[21-23]. However, HIV-1 can evolve increased neutralization sensitivity to broadly neutralizing monoclonal antibodies (bnmAb) and that the spectrum of neutralization capacities by bnmAb can be broader when studied in longitudinal analysis [24].

Our previous study showed that the neutralizing antibody response and the prevalence of naturally occurring cross-reactive neutralizing activity in chronically HIV-1 subtype B’ infected FPDs [25]. In this study, we aimed to determine the intra- and inter-subtype neutralization sensitivity (i.e., the sensitivity of an HIV-1 subtype to the antibodies from patients infected by the same or different HIV-1 subtype, respectively) of HIV-1 B’, which may serve as a basis for designing an AIDS vaccine to prevent HIV-1 subtype B’ infection.

## Results

### General characteristics of the study subjects and viral subtypes

Viruses were isolated from the peripheral blood mononuclear cells (PBMCs) of 12 HIV-1-infected patients, who are FPDs from Anhui province of China. The subjects’ mean age was 40 years (30–52 years), 6 (50%) of them were woman. The mean CD4^+^ and CD8^+^ T cell counts were 289 (48–561) and 1016 (468–1937) per μl of whole blood, respectively. The mean plasma viral load (VL) was 4.95 (3.86-5.74) log_10_ HIV-1 RNA copies per ml.

**Figure 1:**
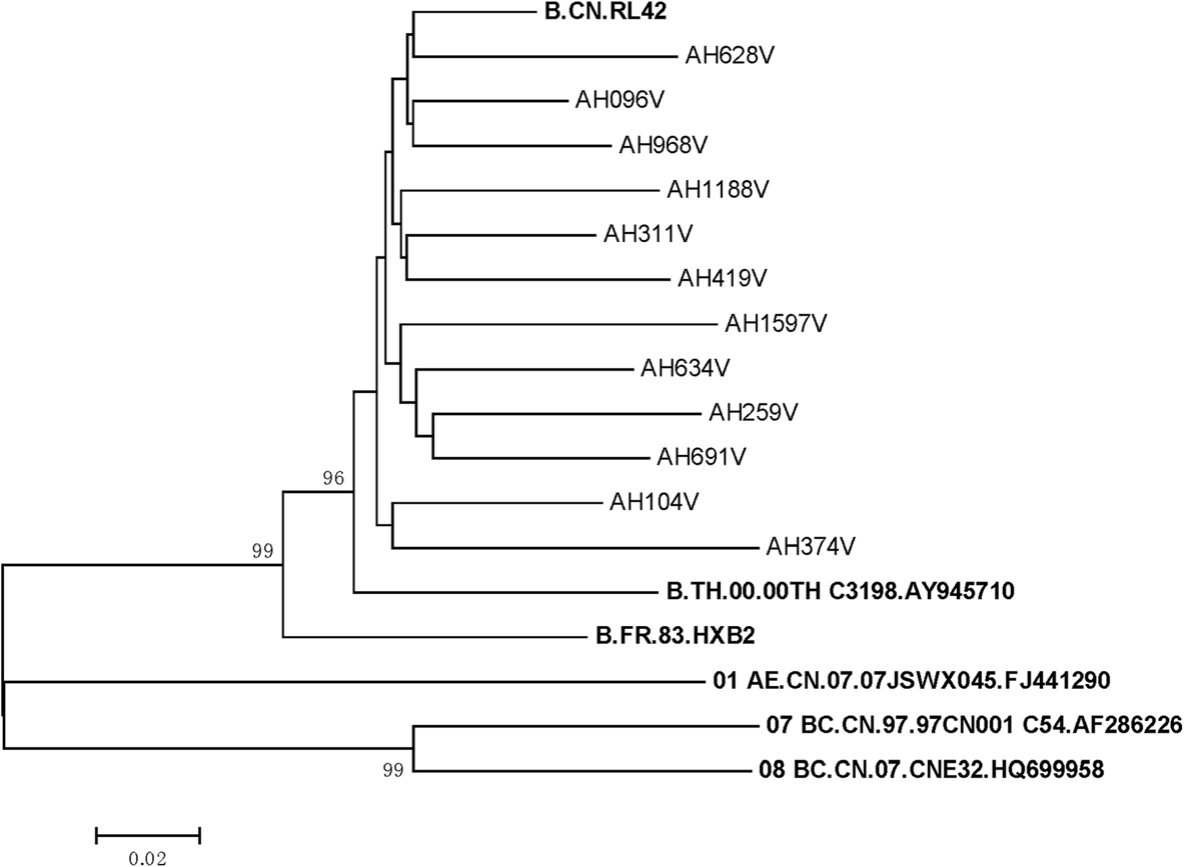
**Neighbor-joining tree depicting the genetic relationship among the HIV-1 isolates.** A number of commonly used reference sequences for classifying HIV-1 subtypes (in bold) and CRFs were included. All the isolates from Anhui (AHxxxV) were close to B.FR.HXB2 (HIV-1 subtype B) and closer to B.CN.RL42 (Thai B’) based on the phylogenetic analysis of gp120 gene.

**Figure 2:**
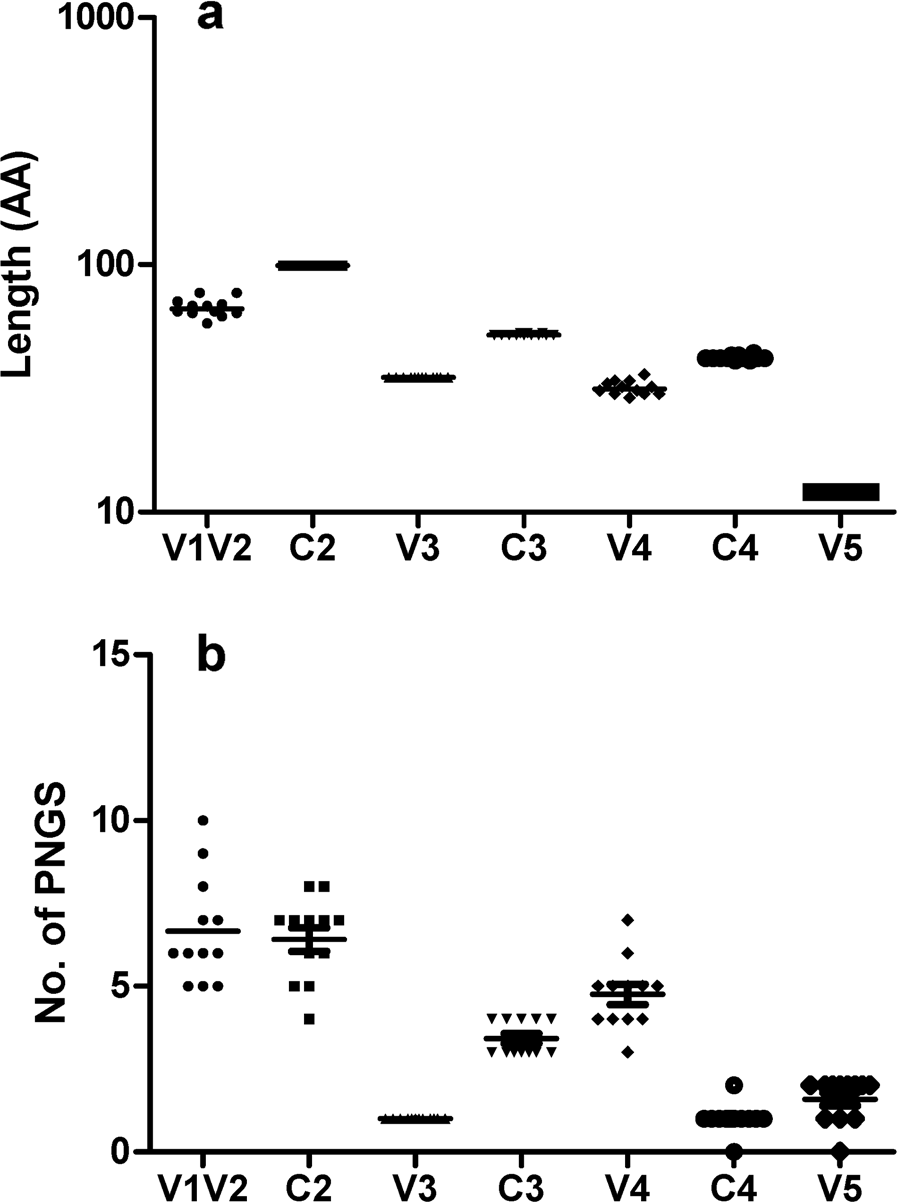
**Length of the gp120 V1-V5 subregions and the number of PNGS in the gp120 V1-V5 subregions.** The values on the *y*-axis indicate the length in amino acids (AA) (**a**) and the number of PNGS (**b**) in the gp120 V1-V5 subregions of 12 HIV-1 subtype B’ isolates.

Phylogenetic analysis of the gp120 region gene sequence confirmed that all HIV-1 isolates were HIV-1 subtype B’. The phylogenetic tree showed that they were close to B.FR.HXB2 (HIV subtype B) and closer to B.CN.RL42 (Thai B’, a clade of HIV-1 B) (Figure 1). The amino acid (AA) lengths and numbers of PNGS in the V1-V5 regions were analyzed by comparing their HIV-1 gp120 sequences. The V1V2 (67±1.6) and V4 (32±0.6) regions displayed a relative heterogeneity in lengths, whereas the V3 loop (35±0), V5 (12±0), C2 (99±0), C3 (52±0.1) and C4 (42±0.2) regions showed little length variation (Figure 2a). The number of PNGS ranged from 22 to 28 in V1-V5 regions with little change in the number of PNGS in the V3, C3, C4 and V5 regions except for V1V2, C2 and V4 (Figure 2b).

### Neutralization sensitivity of HIV-1 B’ isolates

**Figure 3:**
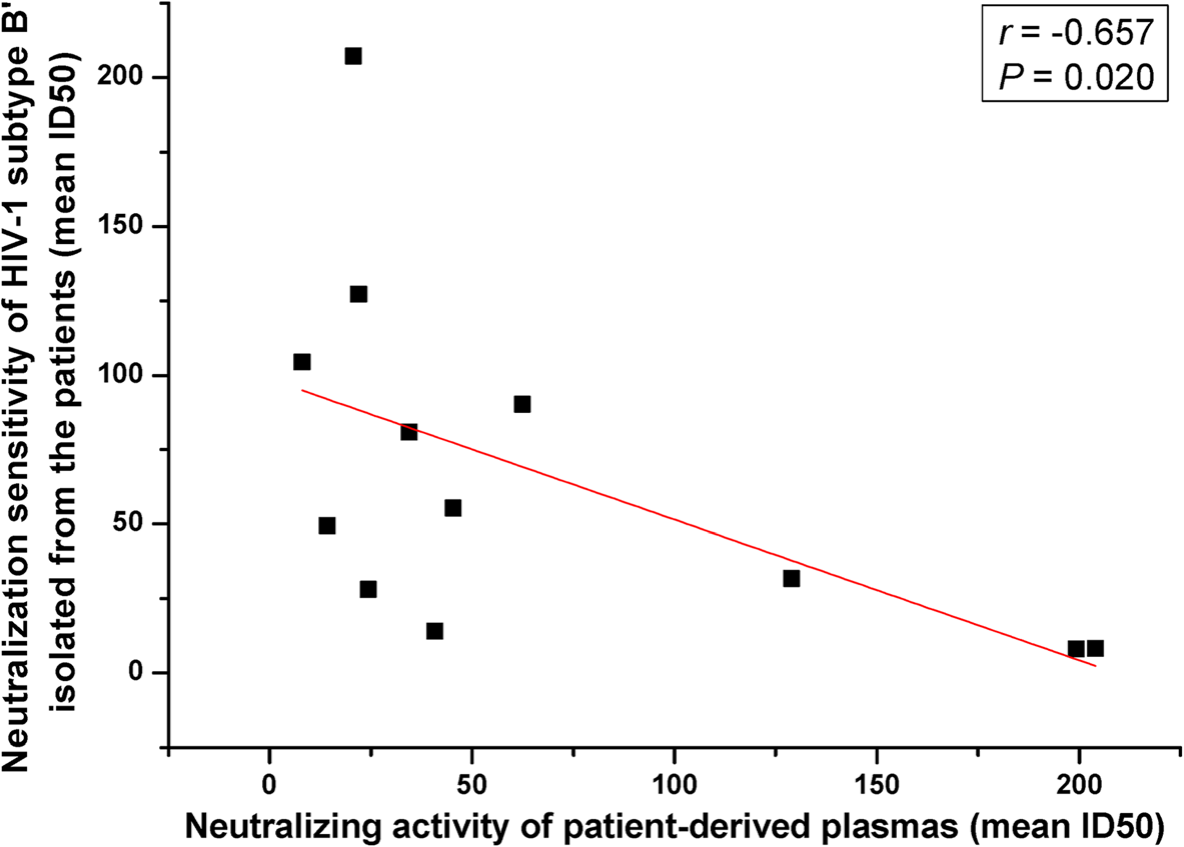
**Correlation between the neutralization sensitivity of the HIV-1 B’ isolates and the neutralizing activity of the plasmas from HIV-1 B’-infected patients.** The intra-subtype neutralization sensitivity of the HIV-1 B’ isolates is inversely correlated with the neutralizing activity of the plasmas from HIV-1 B’-infected patients against these isolates. *P* values (two-sided) and *r* values are based on Spearman’s rank test.

The above 12 HIV-1 B’ isolates were tested for their intra- and inter-subtype neutralization sensitivities to the plasmas from patients infected by the same subtype (HIV-1 B’) and different subtype (HIV-1 CRF07_BC), respectively. As shown in Table 1, most HIV-1 B’ isolates exhibited high intra-subtype neutralization sensitivity to the heterologous plasmas from the HIV-1 B’-infected patients with mean ID50 titer (1/x) of about 67, while they were resistant to most autologous plasmas with mean ID50 titer of ≤ 8 (shown as “―” in the table). Interestingly, the neutralization sensitivity of HIV-1 B’ isolates was inversely correlated with the neutralizing activity of the plasmas from the HIV-1 B’-infected patients (Figure 3; *r* = −0.657, *P* = 0.020). Some isolates, such as AH1597V and AH311V exhibited high neutralization sensitivity to most plasmas from the HIV-1 B’-infected patients with mean ID50 titers (1/x) of about 104 and 207, respectively, while their corresponding plasmas, AH1597P and AH311P, were not effective in neutralizing most of these HIV-1 B’ isolates with mean ID50 titers (1/x) of about ≤ 8 and 21, respectively. In contrast, some of the isolates, such as AH259V and AH691V, had low neutralization sensitivity to most plasmas from the HIV-1 B’-infected patients, with mean ID50 titers (1/x) about ≤ 8 and 8.2, respectively. However, their corresponding plasmas (i.e., AH259P and AH691P) were effective in neutralizing most of these HIV-1 B’ isolates, with mean ID50 titers (1/x) about 199 and 204, respectively (Table 1). The sensitivity of these isolates to neutralization by VRC01 [26] that a well known broadly neutralizing monoclonal antibodies (bnmAb) included as a control were studied. It is showed that the neutralization sensitivity (IC_50_) of AH259V, AH691V and AH096V to VRC01 were more than a concentration of 10μg/ml (Table 1). The viral inter-subtype neutralization sensitivity of the HIV-1 B’ isolates to the plasmas derived from 8 patients infected by HIV-1 CRF07_BC was determined. As shown in Table 2, most of the HIV-1 B’ isolates displayed low inter-subtype neutralization sensitivity to the plasmas from those patients with mean ID50 titer (1/x) of about 22. All these results suggest that some viruses able to induce broad neutralizing antibody response gain machinery to protect themselves from the attack by the neutralizing antibodies.

**Table 1:** Neutralization sensitivity of the HIV-1 B’ isolates to the plasmas from HIV-1 B’ infected patients

**Virus (co-receptor usage)**	**Neutralizing activity (ID50 titer, 1/x) of plasma from HIV-1 B’ infected patients**													**VRC01**^**§ **^**IC50(μg/mL)**	**ADS-J1**^**# **^**(% Inhibition)**
	**AH096P**	**AH104P**	**AH259P**	**AH311P**	**AH374P**	**AH968P**	**AH419P**	**AH628P**	**AH634P**	**AH691P**	**AH1188P**	**AH1597P**	**Mean**		
AH096V (R5)	***11***	21	204	38	57	77	21	90	―	50	―	―	49.4	>10	63.9
AH104V (X4R5)	18	***―***	119	14	12	―	16	20	―	98	―	―	28.1	1.107	46.9
AH259V ( R5)	―	―	***―***	―	―	―	―	―	―	―	―	―	―	>10	77.0
AH311V (X4R5)	22	40	≥ 648	***―***	18	58	214	≥ 648	166	≥ 648	―	―	207.2	1.473	92.2
AH374V (X4R5)	19	19	122	19	***―***	―	82	50	―	501	127	―	80.9	0.081	94.2
AH968V (R5)	26	78	167	20	39	***―***	50	159	21	487	20	―	90.3	0.693	86.4
AH419V (R5)	―	―	78	―	―	10	***―***	―	―	―	―	―	14.0	0.601	82.2
AH628V (R5)	10	10	148	―	15	112	―	***―***	―	38	―	―	31.8	ND	86.6
AH634V (R5)	―	―	140	―	65	120	20	―	***―***	263	―	―	55.3	ND	83.0
AH691V (R5)	―	―	―	―	―	10	―	―	―	***―***	―	―	8.2	>10	81.3
AH1188V (X4R5)	14	50	474	88	132	231	24	240	17	239	***―***	―	127.1	0.320	63.4
AH1597V (R5)	20	35	275	21	44	100	30	300	275	100	45	***―***	104.4	ND	94.7
Mean	14.3	24.4	199.3	20.7	34.5	62.5	40.8	129	45.3	204.0	22.0	―			
SF33 (X4R5)*	206	156	≥ 648	≥ 648	≥ 648	80	622	441	187	399	80	≥ 648			
Panel of Env-pseudotyped viruses^&^															
Clade B (n=8)	28.9	48.8	103.1	28.9	82.0	102.9	84.5	143.0	61.6	359.4	36.8	―	91.7		
Clade C (n=4)	30.5	27.5	140.5	30.5	28.3	59.0	27.5	96.0	28.3	136.0	59.5	―	57.0		
Clade A (n=4)	28.3	―	108.0	28.3	56.0	33.5	109.0	146.3	26.5	123.0	42.3	―	61.8		

The plasmas were tested at a series of 3-fold dilutions ranging from 1:8 to 1:648. “―” means that the neutralizing titer is “≤ 8”. For calculation, the ID50 titers (1/x) of ≤ 8 and ≥ 648 were treated as 8 and 648, respectively. The neutralizing titers of plasmas against the autologous virus are marked in **bold** and *italics*. *A laboratory-adapted HIV-1 strain was included as a control. ^&^ The sensitivity of 12 plasmas were tested against Env-pseudotyped viruses of reference viruses panels [27-29], which displayed mean of ID50 titers (1/x) in the table, and “―” means that the neutralizing titer is “≤ 20” in this section (detail showed in Additional file 1: Table S1). ^§^VRC01 (broadly neutralizing monoclonal antibodies) was included as a control [26]. ^#^ADS-J1 (HIV-1 entry inhibitor) at 10 μg/ml was included as a control of the HIV-1 inhibitor [30,31].

**Table 2:** Neutralization sensitivity of the HIV-1 B’ isolates to the plasmas from HIV-1 CRF07_BC infected patients

**Virus (co-receptor usage)**	**Neutralizing activity (ID50 titer, 1/x) of plasma from HIV-1 CRF07_BC infected patients**								
	**XJ256P**	**XJ257P**	**XJ0135P**	**XJ6291P**	**XJ6371P**	**XJ0793P**	**XJ1353P**	**XJ1981P**	**Mean**
AH096V (R5)	―	―	10	10	28	―	―	―	11.0
AH104V (X4R5)	―	―	―	―	13	―	―	―	8.6
AH259V ( R5)	―	―	―	―	―	―	―	―	―
AH311V (X4R5)	―	―	10	―	27	―	―	―	10.6
AH374V (X4R5)	―	―	―	368	―	―	―	―	53.0
AH968V (R5)	―	―	―	―	35	―	―	―	11.4
AH419V (R5)	―	―	―	―	―	―	―	―	―
AH628V (R5)	―	―	―	―	―	―	13	―	8.6
AH634V (R5)	20	―	―	11	18	―	24	―	13.1
AH691V (R5)	16	―	―	―	―	≥ 648	41	22	94.9
AH1188V (X4R5)	16	20	―	―	20	10	29	24	16.9
AH1597V (R5)	18	―	54	10	21	―	17	―	18.0
Mean	11.2	9.0	12.2	38.6	16.8	61.5	15.0	10.5	
SF33(X4R5)*	160	≥ 648	―	―	236	304	56	316	

The plasmas were tested at a series of 3-fold dilutions ranging from 1:8 to 1:648. “―” means that the neutralizing titer is “≤ 8”. For calculation, the ID50 titers (1/x) of ≤ 8 and ≥ 648 were treated as 8 and 648, respectively. *A laboratory-adapted HIV-1 strain was included as a control.

12 plasmas from HIV-1 B’-infected patients were tested against a panel of 16 Env-pseudotyped reference viruses, including eight clade B strains (SVPB6, SVPB8, SVPB11, SVPB13, SVPB14, SVPB16, SVPB17 and SVPB19) [27], four C strains (SVPC5, SVPC10, SVPC13 and SVPC16) [28] and four A strains (HIV-Q461, HIV-Q769, HIV-Q259 and HIV-Q842) [29]. We found that clade B reference viruses were more easily neutralized by plasmas from HIV-1 subtype B’ infected patients (mean ID50 titer (1/x): 91.7) as compared to clade C (mean ID50 titer (1/x): 57.0) and clade A reference viruses (mean ID50 titer (1/x): 61.8). 5 of 12 subtype B’ plasma samples showed broad neutralization activity against more than 80% of reference viruses, especially, the plasma samples AH259P, AH691P and AH628P. The three plasma samples were appeared high neutralization activity, whereas AH1597P failed to neutralized any of the panel of viruses tested, which was alike to the result of plasma samples against autologous or heterologous isolates from the HIV-1 B’-infected patients (Table 1 and Additional file 1: Table S1).

As shown in Table 1, 8/12 viruses (66.67%) used co-receptor CCR5 for cell entry, while the remaining four (33.33%) used both CCR5 and CXCR4. There was no difference between the R5 and R5X4 viruses in intra-subtype neutralization sensitivity (*P* = 0.126) or inter-subtype neutralization sensitivity (*P* = 0.349).

### Potential correlation of neutralization sensitivity of the HIV-1 B’ isolates with the clinical and gp120 features

**Figure 4:**
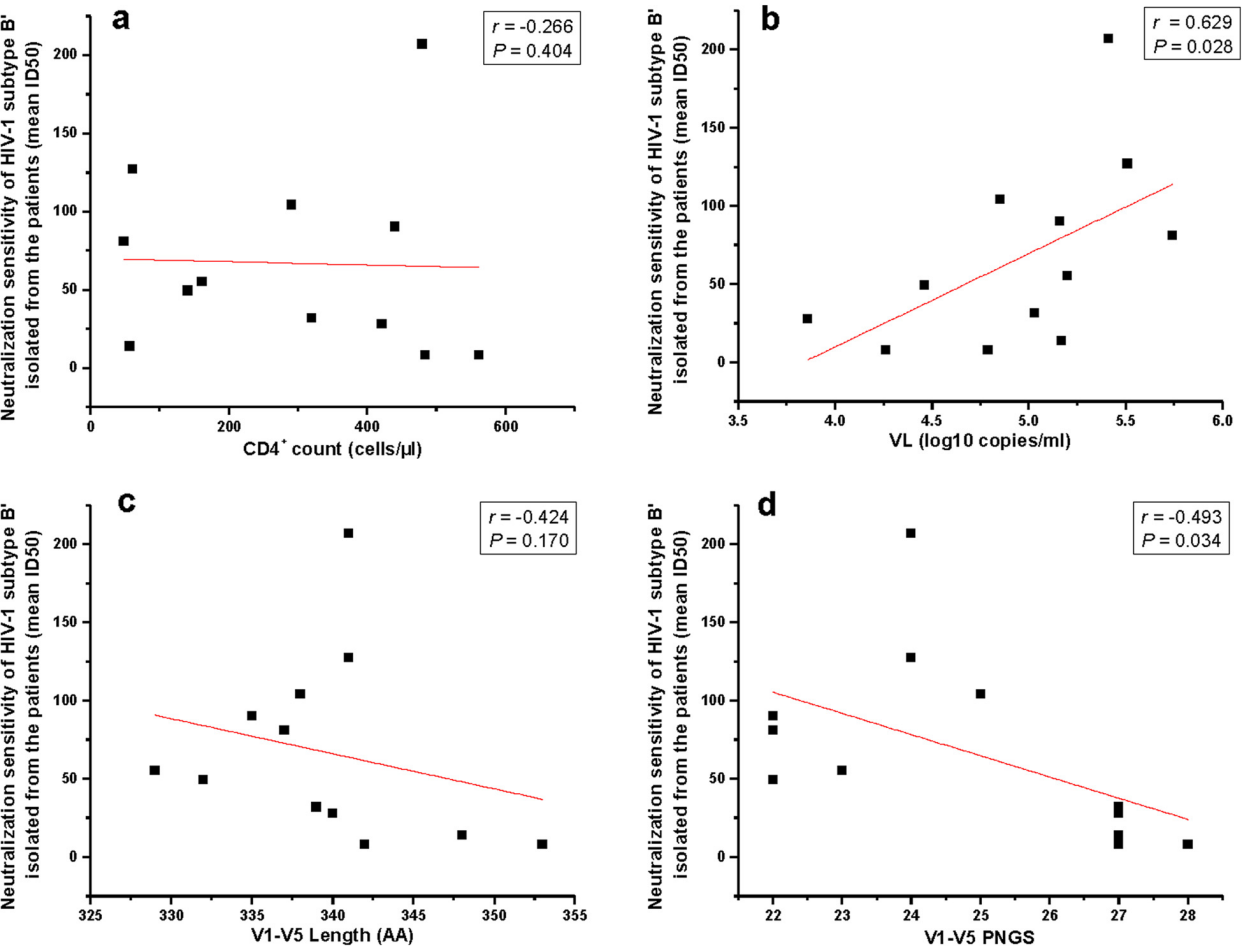
**Correlation between neutralization sensitivity of the HIV-1 B’ isolates and both clinical and gp120 features.** The potential correlation between the neutralization sensitivity of the HIV-1 B’ isolates and CD4^+^ T cell count (**a**), viral load (**b**), length of the gp120 V1-V5 subregions (**c**), and the number of PNGS in the V1-V5 subregions in gp120 (**d**) was analyzed by Spearman rank order correlation test. The values on the *y*-axis indicate the neutralization sensitivity (mean ID50 titer) of 12 HIV-1 subtype B’ isolates. *P* values (two-sided) and *r* values are based on Spearman’s rank test.

The potential correlation between neutralization sensitivity of the HIV-1 B’ isolates and the clinical features, such as CD4^+^ T cell count and viral load (VL), or the gp120 features, such as length of the gp120 V1-V5 sub-regions and the number of PNGS in the V1-V5 sub-regions of gp120, was analyzed with the Spearman rank order correlation test. We found that the neutralization sensitivity of the HIV-1 B’ isolates was positively correlated with VL (Figure 4b; *r* = 0.629, *P* = 0.028), but inversely correlated with the number of PNGS in the gp120 V1-V5 sub-regions (Figure 4d; *r* = −0.493, *P* = 0.034). No significant correlation was observed between the neutralization sensitivity of the HIV-1 B’ isolates and CD4^+^ T cell count (Figure 4a; *r* = −0.266, *P* = 0.404) or the lengths of the gp120 V1-V5 sub-regions (Figure 4c, *r* = −0.424, *P* = 0.170). These results suggest that the HIV-1 B’ subtype may become resistant to the neutralizing antibodies by increasing the number of PNGS in the gp120 V1-V5 sub-regions.

### Characterization of AH259V and AH259P

**Figure 5:**
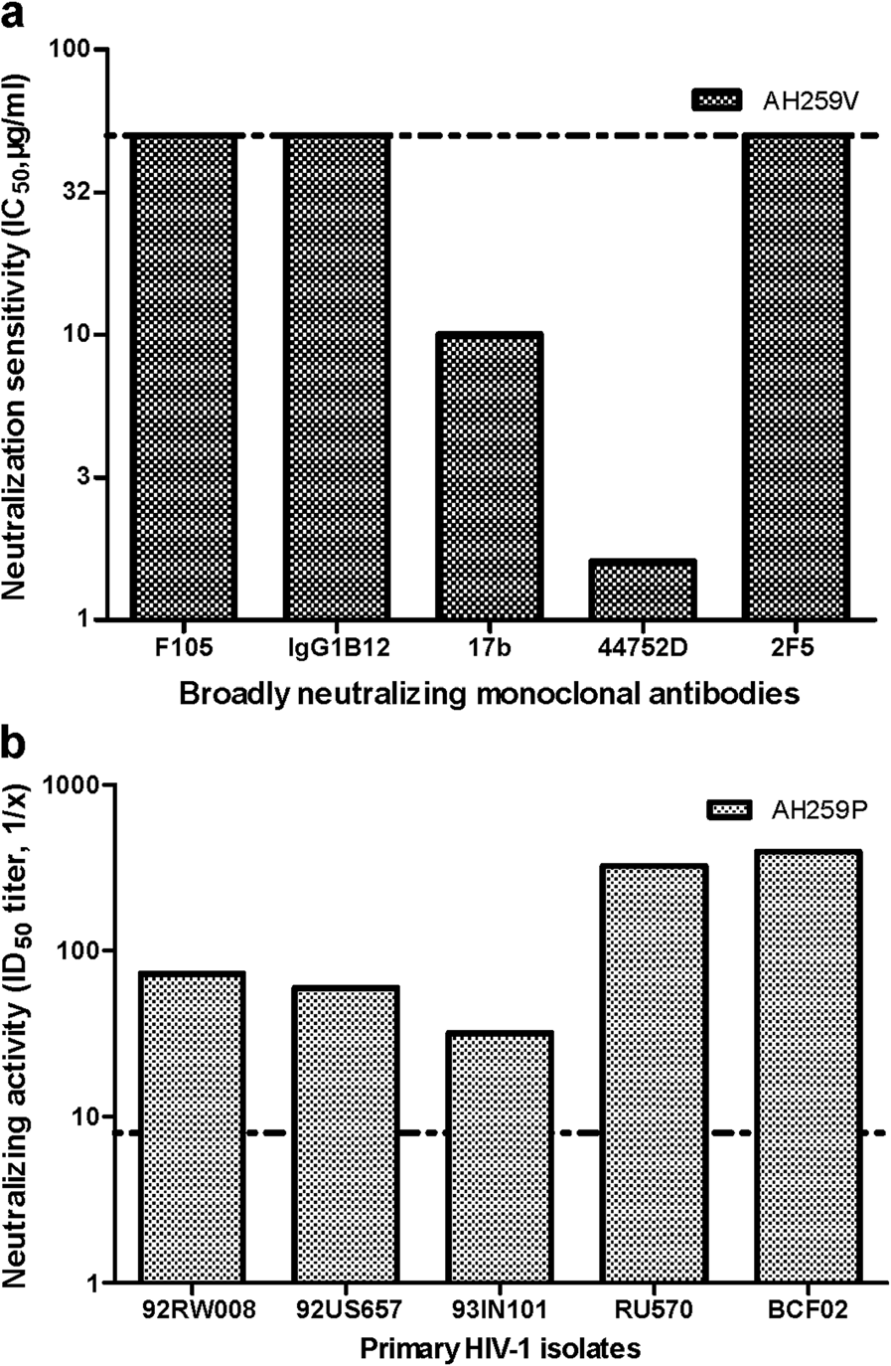
**Neutralization sensitivity of AH259V and neutralizing activity of AH259P.** The neutralization sensitivity of AH259V to the broadly neutralizing monoclonal antibodies **(**F105, IgG1B12, 17b, 447-52D and 2F5) (**a**) and the neutralizing activity of AH259P against the primary HIV-1 isolates, including 92RW002 (subtype A, R5), 92US657 (subtype B, R5), 93IN101 (subtype C, R5), RU570 (subtype G, R5), and BCF02 (group O, R5) (**b**) were determined using a neutralizing assay for HIV-1 infection in PBMCs as described in the Methods. The dotted line indicated that (**a**) the initial concentration of bnmAbs is ranging from 50μg/ml, and IC_50_ >50μg/ml were treated as 50; (**b**) the initial dilutions of plasma is ranging from 1:8, and the ID50 titers (1/x) of < 8 were treated as 8.

As mentioned above, AH259V could not be neutralized by any of the 12 plasma samples collected from the HIV-1 B’-infected patients, whereas AH259P could potently neutralize 10 of the 11 heterologous HIV-1 B’. We then tested the neutralization sensitivity to several other known bnmAbs, including F105 [32], IgG1B12 [33], 17b [34], 44752D [35] and 2F5 [36]. We found that F105, IgG1B12 and 2F5 failed to neutralize AH259V at a concentration of 50μg/ml, whereas 17b and 447-52D can neutralize AH259V with the values of 10 and 1.6μg/ml, respectively (Figure 5a). Subsequently, we tested the neutralizing activity of the plasma AH259P against several primary HIV-1 isolates of different subtypes, including 92RW002 (subtype A), 92US657 (subtype B), 93IN101 (subtype C), RU570 (subtype G), and BCF02 (group O). We observed that the nAbs in AH259P were effective in neutralizing all HIV-1 strains tested with ID50 titers (1/x) in the range of 32–396 (Figure 5b). These results suggest that although AH259V virus has low intra-subtype neutralization sensitivity, it can be neutralized by some known bnmAbs and the nAbs in AH259P are effective in neutralizing infection by the primary HIV-1 isolates with different subtypes. Using PL-ELISA, the epitope specificities of antibodies in AH259P were analyzed with overlapping synthetic peptides covering the sequences of the entire gp120 and both the extracellular and transmembrane domains of gp41 (data not shown). It is showed that the antibodies significantly bound to five peptides, including p27 (gp120 C2 region), p38 (gp120 C3 region), p49 (gp120 C5 region), p64 (gp41 TM region) and p66 (gp41 TM region). However, these epitopes may not be the targets for the neutralizing antibodies since most of the neutralizing epitopes in HIV-1 Env are conformational, rather than linear [37]. Further characterization of AH259V and AH259P is warranted.

## Discussion

In the present study, we evaluated the intra-subtype neutralization sensitivity of HIV-1 B’ isolates to the neutralization antibodies in the plasmas derived from patients from whom the viruses were isolated. We demonstrated that these isolates possess high intra-subtype neutralization sensitivity to the heterologous plasmas, but very low intra-subtype neutralization sensitivity to the autologous plasmas, which is consistent with the results from our previous study on the HIV-1 CRF07_BC recombinant [38]. These findings suggest that most HIV-1 B’ viruses can induce neutralization response, but they become resistant to autologous neutralizing antibodies, most likely by the immune escape mechanism developed during disease progression. By analyzing the neutralizing activity of consecutive patient plasma against the sequentially obtained autologous virus, Arendrup et al. [39] found that the development of neutralization to the primary HIV-1 isolates was accompanied by emergence of escape virus with corresponding reduced neutralization sensitivity to autologous plasma. This may explain the apparent failure of the immune system to control HIV infection. In this study, we also showed that HIV-1 B’ isolates exhibit low inter-subtype neutralization sensitivity to the antibodies in the plasmas derived from the HIV-1 CRF07_BC-infected patients (Table 2), which may caused from low cross-reactive neutralizing activity in different subtype or the different neutralization potency in same subtype.

According to previous reports, the antibody response matures and becomes cross-neutralizing against divergent HIV-1 in up to 10% of HIV-1-infected patients, as shown in neutralization assays [40,41]. Interestingly, we found that the antibodies in some HIV-1 B’-infected patients have potent cross-neutralizing activity. 5 of 12 subtype B’ plasma samples showed broad neutralization activity against more than 80% of Env-pseudotyped reference viruses that covered HIV-1 clade B, C and A. And specially, the antibodies in AH259P could potently neutralize infection of PBMCs by a series of primary HIV-1 isolates with different subtypes (Figure 5b), while the AH259V virus was shown to be sensitive to neutralization by two known bnmAbs in this study (Figure 5a). VRC01 failed to neutralize AH259V, AH691V and AH096V at a concentration of 10μg/ml (Table 1). It is reported that many HIV-1 strains in China were resistant to PG9, PG16 and VRC01, which known to possess exceedingly high potency and breadth against diverse viruses from outside China [42]. These findings suggest that although most HIV-1 B’ viruses have low inter-subtype neutralization sensitivity, some of these primary HIV-1 isolates are able to induce both intra- and inter-subtype cross-neutralizing antibody responses.

It is reported that X4 HIV-1 viruses are generally more sensitive than R5 viruses to neutralizing antibodies [43]. Most HIV-1 B’ primary HIV-1 isolates collected from the FPDs were R5 viruses, while a few of them were dual-tropic R5X4 viruses. In our study, no significant difference in intra- and inter-subtype neutralization sensitivity between the R5 and R5X4 viruses was observed, suggesting that these R5X4 viruses may predominantly use CCR5 co-receptor for infection.

Positive correlation was shown between neutralization sensitivity of the HIV-1 B’ isolates and viral load in patients from whom the viruses were isolated, but no significant correlation between viral neutralization sensitivity and CD4^+^ T cell count in these patients was observed. Notably, the neutralization sensitivity of HIV-1 B’ isolates is inversely correlated with neutralizing activity of plasmas from the HIV-1 B’-infected patients (Figure 3), and with the number of PNGS in the V1-V5 sub-regions of gp120 (Figure 5d). This finding agrees with the reports of others showing that the decreased neutralization sensitivity of HIV-1 isolates results from increased glycan numbers in the viral *env* used to build up a “glycan shield” to protect the virus from attack by neutralizing antibodies [44,45]. Interestingly, increase in the number of PNGS is generally located in the gp120 variable regions of the neutralization-resistant isolates. AH259V, for instance, has 10 PNGS in V1-V2 region, while other viruses (e.g., AH1597V) with less resistance have 5 PNGS in the same region.

Dhillon et al. [46] characterized the neutralization antibody present in the plasma of three individuals infected with subtype A and B viruses and found that the highly conserved neutralizing epitope in gp41 and highly variable V3 loop in gp120 may not account for the broad neutralizing activity, while the CD4 binding site in gp120 may be responsible for eliciting the neutralization antibody. We will use similar approaches to further investigate how the HIV-1 subtypes predominant in China induce intra-subtype neutralizing antibody response and protect themselves from attack by the autologous neutralizing antibodies. These studies may provide a sound scientific platform for the rational design of vaccines that induce antibody responses against the HIV-1 isolates circulating in China.

## Conclusions

The results from this study indicate that the HIV-1 B’ clinical isolates retain their neutralization sensitivity to the antibodies in the heterologous plasmas, but become resistant to those in autologous plasmas. Their neutralization sensitivity is inversely correlated with the neutralizing activity of nAbs from the patients infected by the same subtype of HIV-1 and with the number of PNGS in the viral gp120. The HIV-1 B’ clinical isolates lack the inter-neutralization sensitivity to the neutralizing antibodies from the patients infected by the HIV-1 CRF07_BC recombinant. These findings suggest that the HIV-1 B’ viruses may mutate under the immune pressure, thus becoming resistant to the autologous nAbs, possibly by changing the number of PNGS in the V1-V5 region of the viral gp120.

## Methods

### Study population

The study population was selected from a multicenter AIDS Cohort Study in China in 2005 and consisted of pre-selected HIV-1-infected patients, who were naive to antiretroviral therapies. All the patients had a self-reported history of paid blood donation of more than 10 years. The blood samples from 12 of these cohort patients were collected. Whole blood was used for CD4^+^ and CD8^+^ T cell counts and peripheral blood mononuclear cells (PBMCs) were isolated by Ficoll-Paque gradient (Amersham Biosciences, Uppsala, Sweden) for HIV-1 isolation. Plasmas were separated by centrifugation from the citrated dextrose-treated blood samples and stored at −20°C until use for detection of neutralizing antibodies. We also collected plasmas from the HIV-1 CRF07_BC-infected IDUs in Xinjiang Uyghur Autonomous Region of China [38] as controls. This study was approved by the Institutional Research Ethics Community of Chinese Center for Disease Control and Prevention (IRB00002276), and all subjects signed informed consent to participate in the research study forms prior to blood and data collection.

### Detection of viral load (VL), CD4^+^ and CD8^+^ T cell count

Plasma VL was measured using the HIV-1 nucleotide fluorescence quantitative assay kit (BD Biosciences, Franklin Lakes, NJ) according to the manufacturer’s instructions, which has a lower detection limit (LDL) of 500 copies/ml. The average CD4^+^ and CD8^+^ T lymphocyte counts were assessed by flow cytometry analysis (BD Biosciences) [47].

### Isolation of HIV-1 from patients’ PBMCs

Primary HIV-1 viruses were isolated from PBMCs as previously described [39,48]. Briefly, PBMCs isolated from HIV-1-infected individuals were co-cultured with phytohemagglutinin (PHA)-stimulated PBMCs obtained from two or more HIV-1-seronegative healthy donors. The cell cultures were maintained for 4 weeks in RPMI 1640 medium (Gibco) containing 20 U/ml of recombinant interleukin-2 (IL-2), 1% penicillin and streptomycin, 2 mM glutamine, and 10% fetal bovine serum (FBS). Culture medium was changed twice a week. HIV-1 p24 was quantified using a commercial enzyme-linked immunosorbent assay (ELISA) kit (BioMerieux, France) once a week for 4 weeks. The viruses in the culture supernatants with p24 > 2 ng/ml were harvested, titrated, and stored in liquid nitrogen until used.

### Amplification and genetic analysis of HIV-1 gp120 region

Viral RNA was extracted from the isolated viruses using a QIAamp® Viral RNA Mini Kit (Qiagen Inc., Chatsworth, CA) according to the manufacturer’s protocol. The region coding gp120 was amplified from viral RNA by TaKaRa’s One Step RNA PCR Kit (AMV). An outer reaction was performed with the primers ES3 (5^′^-GAATAAGAGAAAGAGCAgAAGA-3^′^, 7219–7240) and eas1 (5^′^-CTAGGAGCTGTTGATCCTTTAGGTA-3^′^, 9005–9028). The amplification was run at 50°C for 30 min, and 94°C for 2 min followed by 40 cycles at 94°C for 30s, 62°C for 30 s (−0.3°C/cycle) and 72°C for 2 min, with a final extension of 72°C for 10min. A nested reaction was then performed with the primers ES4 (5^′^-CAGAAGACAGTGGCAATGAGA-3^′^, 7234–7254) and eas2 (5^′^-GCCTGTACCGTCAGCGTTATT -3^′^, 8850–8870). The amplification was run for 3 cycles at 94°C for 3min, 55°C for 50s, and 72°C for 90s, then followed by 30 cycles at 94°C for 30s, 55°C for 50s and 72°C for 90s, with a final extension of 72°C for 10min (Gene Amp PCR System 9700, Applied Biosystems, Foster City, California, USA). PCR products were gel-purified by using a QIAquick Gel Extraction Kit (QIAGEN, Valencia, CA), then sequenced on an ABI 3770 Sequencer (Applied Biosciences). Chromatograms were examined manually for the presence of double peaks indicative of two templates per sequencing reaction.

Sequences were aligned using GeneCutter (http://www.hiv.lanl.gov/content/sequence/GENE_CUTTER/cutter.html) and Clustal W together with selected subtypes/CRFs and then additionally edited by hand when needed. Phylogenetic analysis was performed using the neighbor-joining method in MEGA5, and the reliability of the branching orders was tested by bootstrap analysis of 1,000 replicates [49]. The length of V1 to V5 (V1-V5) in the gp120 region was analyzed using BioEdit software (version 7.1.3). Potential N-linked glycosylation sites (PNGS) in V1-V5 were predicted using the N-GLYCOSITE web tool from the Los Alamos HIV database [44].

### Determination of viral co-receptor usage

The viral co-receptor usage of the primary HIV-1 isolates was determined as previously described [50]. Briefly, GHOST cells that express CD4 and CXCR4 (Ghost-X4) or CCR5 (Ghost-R5) were seeded in 24-well plates at the density of 1×10^5^ cells/well overnight. When about 70% confluent mono-layers, GHOST cells were infected with virus stocks (200μl/well) containing 8 μg/ml DEAE-dextran, which enhance the infective efficiency. After 48 hours, cells were harvested and analyzed with flow cytometey (Elite ESP, Beckman Coulter, Germany). Approximately 10-fold shifts in mean GFP fluorescence of infected cells over uninfected cell were considered as positive for the corresponding co-receptor [51]. The Ghost-R5 and Ghost-X4 cells infected with HIV-1_SF33_ (R5X4), HIV-1_Ba-L_ (R5) and HIV-1_IIIB_ (X4) were positive controls and the cells without HIV-1 infection were negative control.

### NAb assay in TZM-bl cells

Neutralizing activities of plasma samples and bnmAbs against isolates by a sensitive HIV-1 neutralization assay using TZM-b1 cells were performed as previously described [51,52]. Briefly, a primary HIV-1 isolate (200 TCID_50_) was pre-incubated in triplicate with a series of 3-fold diluted plasma or antibody, followed by incubation for 1h at 37°C. Plasma from HIV-1-seronegative healthy blood donors was included as control. TZM-b1 cells (1×10^4^ cells) in the presence of DEAE-dextran (15 μg/ml) were added to each well. After 48h incubation, 150 μl culture medium was removed from each well and 100μl luciferase reporter gene assay system reagent was added (Bright-Glo; Promega). Following a short incubation (minimum of 2 min), 150 μl lysate from each well was transferred to 96-well black solid plates for measurement of luminescence in a luminometer (PerkinElmer Life Sciences). Mock infected cells were used to determine background luminescence. HIV-1 Env-pseudotyped virus preparation, titration and nAbs assay in TZM-bl cells were as described previously [25]. The neutralizing activity was reported as the reciprocal of dilution (1/x) of plasma required to confer 50% inhibition (ID50) of infection by the virus tested.

### Neutralization of infection by primary HIV-1 isolates in PBMCs

The neutralizing sensitivity of A259V to the broadly neutralizing monoclonal antibodies and the neutralizing activity of A259P against primary HIV-1 isolates of different subtypes were determined as previously described [53]. PBMCs were isolated from health blood donor by standard density gradient centrifugation using Histopaque-1077 (Sigma Chemical Co.). The cells were placed in a 75 cm^2^ plastic flask and incubated at 37°C for 2 hours. The nonadherent cells were collected and resuspended at 5×10^6^ in 10 ml RPMI-1640 medium containing 10% FBS, 5μg/ml PHA (Sigma) and 100 U/ml IL-2 (Sigma), followed by incubation at 37°C for 3 days. The PHA-stimulated cells were infected with corresponding primary HIV-1 isolates at 0.01 multiplicity of infection (MOI). Culture media were changed every 3 days. The supernatants were collected 10 days post-infection and tested for p24 antigen by ELISA as previously described [53]. The percentage of inhibition of p24 production and the concentration for 50% inhibition (IC_50_) was calculated as described before [52].

### Linear antibody epitope mapping

The linear epitopes for the antibodies in AH259P were mapped with overlapping synthetic peptides derived from the sequence of the Env of SHIVchn19 (obtained from the NIH Research and Reference Reagent Program) using PLL-ELISA as previously described [54]. Briefly, the peptides that were diluted to a concentration of 10μg/ml in PBS were added to the wells (50μl/well) coated with PLL (poly-L-leucine, 30–70 kDa, Sigma Aldrich), followed by incubation at room temperature for 1 h and washed with PBS. After addition of 1% glutaraldehyde (Sigma Aldrich), the plate was incubated at room temperature for 15 min and washed twice with PBS. The test plasma that were diluted at 1:400 in PBS containing 5% skimmed dry milk were added into the plates in duplicate and incubated at 37°C for 1 h, followed by addition of HRP-linked anti-human IgG and OPD substrate, sequentially. The OD value for each well was read with an automated plate reader at 450 nm. An OD value greater than two-fold average OD value of background was considered as positive.

### Statistical analysis

Correlations between neutralization sensitivity and variables were analyzed using a Spearman’s rank test (SPSS software package, version 17.0), and a *P* value less than 0.05 was considered statistically significant.

## Competing interests

The authors declare that they have no competing interests.

## Authors’ contributions

LM and YS conceived and designed the experiments. YO, JS, YH, LL, WX, KH, and XH performed the experiments. YO, JS, SJ, YS, and LM analyzed the data and wrote the paper. All authors read and approved the final manuscript.

## Supplementary Material

Additional file 1: Table S1 Neutralization activity of 12 plasmas against a panel of 16 Env-Pseudotyped viruses. Click here for file
