# Supplementary material for: Neutralization sensitivity of HIV-1 subtype B’ clinical isolates from former plasma donors in China
Source: Virol J. 2013 Jan 5;10:10. doi: 10.1186/1743-422X-10-10 (PMC3599083; doi:10.1186/1743-422X-10-10)
Supplement: Additional file 1: Table S1 — Neutralization activity of 12 plasmas against a panel of 16 Env-Pseudotyped viruses. [file 1743-422X-10-10-S1.docx]

Table S1. Neutralization activity of 12 plasmas against a panel of 16 Env-Pseudotyped viruses.

| **Panel of Env-Pseudotyped viruses** | **Neutralizing activity (ID50 titer, 1/x) of plasma from HIV-1 B’ infected patients** | | | | | | | | | | | |
| --- | --- | --- | --- | --- | --- | --- | --- | --- | --- | --- | --- | --- |
|  | AH096P | AH104P | AH259P | AH311P | AH374P | AH968P | AH419P | AH628P | AH634P | AH691P | AH1188P | AH1597P |
| **Clade B** |  |  |  |  |  |  |  |  |  |  |  |  |
| SVPB6 | 56 | 118 | 138 | 56 | 110 | 102 | 85 | 111 | 50 | 145 | 84 | ― |
| SVPB8 | ― | 28 | 65 | ― | 43 | 24 | 98 | 132 | 81 | 211 | ― | ― |
| SVPB11 | ― | ― | 108 | ― | 26 | 31 | 85 | 212 | ― | 217 | ― | ― |
| SVPB13 | 33 | 92 | 63 | 33 | 135 | 38 | ― | 126 | ― | 828 | ― | ― |
| SVPB14 | ― | ― | 46 | ― | 50 | 38 | 23 | 151 | 68 | 334 | ― | ― |
| SVPB16 | 42 | 72 | 207 | 42 | 94 | 141 | 150 | 168 | 80 | 312 | 45 | ― |
| SVPB17 | ― | ― | 123 | ― | 104 | 34 | 187 | 116 | ― | 491 | 28 | ― |
| SVPB19 | ― | ― | 75 | ― | 94 | 415 | 28 | 128 | 154 | 337 | 57 | ― |
| Mean | 28.9 | 48.8 | 103.1 | 28.9 | 82 | 102.9 | 84.5 | 143 | 61.6 | 359.4 | 36.8 | ― |
| **Clade C** |  |  |  |  |  |  |  |  |  |  |  |  |
| SVPC5 | ― | ― | 103 | ― | 39 | 32 | ― | 142 | ― | 152 | 134 | ― |
| SVPC10 | 26 | ― | 99 | 26 | 34 | 67 | 30 | 97 | ― | 216 | 50 | ― |
| SVPC13 | 56 | 50 | 149 | 56 | ― | 113 | 40 | ― | 53 | ― | 34 | ― |
| SVPC16 | ― | ― | 211 | ― | ― | 24 | ― | 125 | ― | 156 | ― | ― |
| Mean | 30.5 | 27.5 | 140.5 | 30.5 | 28.3 | 59 | 27.5 | 96 | 28.3 | 136 | 59.5 | ― |
| **Clade A** |  |  |  |  |  |  |  |  |  |  |  |  |
| HIV-Q461 | ― | ― | ― | ― | ― | 23 | 21 | 81 | ― | 120 | ― | ― |
| HIV-Q769 | 53 | ― | 347 | 53 | 44 | 51 | 90 | 227 | 46 | 261 | 109 | ― |
| HIV-Q259 | ― | ― | ― | ― | 65 | 40 | ― | ― | ― | 20 | ― | ― |
| HIV-Q842 | ― | ― | 45 | ― | 95 | ― | 305 | 257 | ― | 91 | ― | ― |
| Mean | 28.3 | ― | 108 | 28.3 | 56 | 33.5 | 109 | 146.3 | 26.5 | 123 | 42.3 | ― |
| Total |  |  |  |  |  |  |  |  |  |  |  |  |
| Mean | 29.1 | 36.3 | 113.7 | 29.1 | 62.1 | 74.6 | 76.4 | 132.1 | 44.5 | 244.4 | 43.8 | ― |

The plasmas were tested at a series of 3-fold dilutions ranging from 1:20. “―” means that the neutralizing titer is “≤ 20”. For calculation, the ID50 titers (1/x) of ≤ 20 were treated as 20.
